# Integration of Advance Information about a Forthcoming Task Switch – Evidence from Eye Blink Rates

**DOI:** 10.3389/fpsyg.2017.00290

**Published:** 2017-02-28

**Authors:** Thomas Kleinsorge, Juliane Scheil

**Affiliations:** Leibniz Research Centre for Working Environment and Human Factors (LG)Dortmund, Germany

**Keywords:** task switching, dopamine, preparation, executive control, eye blink rate

## Abstract

We investigated task switching among four tasks by means of a modified cuing procedure with two types of cues. One type of cue consisted of a standard task cue indicating the next task. In half of the trials, this task cue was preceded by another type of cue that reduced the set of candidate tasks from four to two tasks. In addition, we measured participants’ spontaneous eye blink rates (EBRs) at the beginning, in the middle, and at the end of the experiment. Whereas interindividual differences in mean EBR had no pronounced effect on task switching performance, changes in EBRs during the first half of the experiment significantly modulated the interaction of the effects of the two types of cues. We suggest that changes in EBRs in the early phase of the experiment reflect adaptations of dopaminergic projections serving to integrate advance information about a forthcoming task switch.

## Introduction

Experiments on task switching aim at elucidating the mechanisms underlying the remarkable human ability to adjust cognition and action according to dynamically changing demands (cf. Kiesel et al., 2010, for a review). On a conceptual level, a certain way of interpreting sensory input and acting accordingly is assumed to be implemented by a particular ‘task set,’ and a change of the way sensory input is dealt with is assumed to be accompanied by a reconfiguration of the task set. In a typical task switching experiment, changing demands (or ‘task switches’) are most often induced by presenting external cues signaling the need to adopt a different task set, but they can also be the result of a change in internal conditions or follow an internally represented action plan.

In the vast majority of task switching experiments, switching proceeds among only two tasks. There is also a line of research, devoted to the so-called backward inhibition effect, which for methodological reasons investigates switching among three tasks (cf. Koch et al., 2010). However, there are considerably less studies on switching among four or even more tasks. This neglect of task environments with a larger number of tasks seems to be problematic for several reasons. First, with regard to ecological validity, people are quite often confronted with situations in which more than two action alternatives are available. Second, there is evidence that switching among four tasks exhibits substantial functional differences compared to switching among only two tasks. For example, mere foreknowledge of an upcoming task without explicit cues is much more effective with four as compared to two tasks (e.g., Kleinsorge and Apitzsch, 2012), suggesting that task selection is based on more elaborate task coding in the former as compared to the latter case (cf. Kleinsorge and Scheil, 2015, for details). Third, selection of a certain action often proceeds in a gradual manner, starting from restricting the number of alternative actions to a limited number of candidate actions followed by choosing among the remaining options. Such a situation was instantiated in the present experiment.

On a neurophysiological level, choosing among candidate actions is intimately linked to processes affected by the neuromodulator dopamine. In this respect, two structures are strongly influenced by dopaminergic projections, the prefrontal cortex (PFC) and the basal ganglia (BG). Both structures are heavily interconnected, but the details of their interplay are far from being completely understood. Regarding the PFC, dopamine is assumed to modulate the balance between robust maintenance of representations in working memory and their flexible updating (e.g., Durstewitz and Seamans, 2008). The updating of working memory representations in PFC is also influenced by the BG that are assumed to provide a ‘Go signal’ facilitating such an updating (cf. Frank and O’Reilly, 2006). The generation of this Go signal proceeds along a ‘direct pathway’ that relies mostly on the D1 subtype of dopamine receptors. This direct pathway is complemented by an ‘indirect pathway’ which relies primarily on D2 receptors. The indirect pathway provides a ‘Nogo signal’ that suppresses competing responses. Importantly, while higher levels of dopamine provide excitatory input to the direct pathway, facilitating the generation of a Go signal, high levels of dopamine have inhibitory effects on the indirect pathway, thereby weakening the D2-driven tonic inhibition of competing responses.

Evidence suggests that variations in eye blink rates (EBRs) are intimately linked to dopamine-driven cognitive processes, with higher EBRs reflecting more involvement of dopaminergic processing (cf. Jongkees and Colzato, 2016, for a recent review). In this respect, EBRs are probably mainly related to the D2 receptor system of the BG (cf. Groman et al., 2014). According to the ‘prepare and select’-model of dopaminergic function in the striatum by Keeler et al. (2014), one key functional distinction between the D1-dominated direct pathway and the D2-dominated indirect pathway consists of the independence vs. competitiveness of action representations within corticostriatal connections: Whereas action representations within the direct pathway are shaped by reward association strength in a rather independent manner, action representations within the indirect pathway are subject to lateral inhibition.

Given that our current understanding strongly suggests a key role for fronto-striatal circuits that are modulated by dopamine in the flexible updating and maintenance of the contents of working memory, and assuming that interindividual differences in EBRs reflect variations in the efficiency of parts of these circuits, it makes sense to expect that performance in task switching experiments should correlate with variations in EBRs. Such an expectation is also corroborated by studies showing that administration of the D2 receptor agonist bromocriptine improves task switching performance, with this improvement being prevented by pretreatment with the D2 receptor antagonist sulpiride (van Holstein et al., 2011). In line with this reasoning, there are studies showing that variations in individual EBR indeed correlate with task switching performance. However, this relationship is not as straightforward as one might wish. The currently best established finding, which was originally reported by Dreisbach et al. (2005) and subsequently replicated by Müller et al. (2007) as well as Tharp and Pickering (2011), consists of the observation that high EBRs go along with reduced switch costs when a post-switch target stimulus is associated with a previously not presented feature (color) while a to-be ignored stimulus (distractor) is associated with the previous target feature (‘perseveration condition’). However, when a post-switch target stimulus is associated with a previously to-be ignored feature while a to-be ignored stimulus is associated with a previously not presented feature (‘learned irrelevance condition’), high EBRs go along with increased switch costs, as compared to low EBRs. This interaction might be explained by the assumption that relatively high dopaminergic activity goes along with a novelty bias that aids performance when task-relevant information is associated with a new feature, but impairs performance when the new feature is associated with distracting information (cf. Dreisbach et al., 2005).

While the aforementioned findings strongly suggest an effect of dopaminergic activity on task switching performance driven by novelty, it is quite unusual in typical task switching experiments to associate either relevant or irrelevant information with a novel feature. Rather, in most of these studies all possibly (ir)relevant stimulus features are introduced already from the outset. Typical stimuli in task switching experiments are, for example, letter-digit combinations (e.g., Rogers and Monsell, 1995). During a task switch, letters and digits change their role as targets vs. distractors without any ‘new’ features serving to facilitate or hinder the performance of a switch. In such a situation, in case of a switch the competition between task relevant and irrelevant routes of information processing has to be resolved by either boosting the activation of the currently relevant or by diminishing the activation of a previously relevant but now irrelevant processing route (or by a combination of both). Both of these processes probably rely in part on dopaminergic projections. Furthermore, as outlined above, the ‘prepare and select’-model proposed by Keeler et al. (2014) suggests that while boosting a now-relevant task set may rely more heavily on a D1-mediated signal, D2-mediated processes may be more implicated in the competition by now-irrelevant task sets. This assumption provides the rationale of the present study.

In the present experiment, we employed the double-cue procedure originally introduced by Kleinsorge and Scheil (2015). Participants were asked to switch among four tasks. During a single trial, this set of four tasks may or may not be reduced to a set of only two candidate tasks by a first cue (pre-cue). A second cue (task cue) may or may not designate one of the tasks as the relevant one in advance of the onset of the imperative stimulus. Ultimately, the relevant task is indicated by the task cue presented concurrently with the target stimulus. Thus, the experiment was based on a 2 × 2 design in which the first factor determined whether the relevant task was selected among four or two candidate tasks, and the second factor determined whether the relevant task was selected in advance or only after the presentation of the imperative stimulus. (Whether the task was a task repetition or a switch constituted a third factor). The main finding of the original study of Kleinsorge and Scheil (2015) was that reducing the number of candidate tasks from four to two provided an advantage that affected task switches significantly stronger than task repetitions.

In the present study, we replicated this experiment and measured participants’ EBRs in addition. We reasoned that the facilitation of task switches provided by reducing the number of candidate tasks might have been due to a lower updating threshold induced by lower competition among tasks because of a smaller number of competing tasks. According to the ‘prepare and select’-model proposed by Keeler et al. (2014), such an effect should be located primarily within the D2-pathway of the BG. Based on the assumption that EBRs primarily reflect the dopaminergic activity within that pathway, we expected to observe significant modulations of the effect of reducing the number of candidate tasks by EBRs. However, due to the complexity of dopaminergic modulations of fronto-striatal circuits, we were reluctant to make specific predictions regarding the precise nature of these correlations. On a behavioral level, we expected to replicate our former observations (Kleinsorge and Scheil, 2015) that both the pre-cue and the task cue would result in pronounced reductions of mean response times and switch costs, with our main interest being focused on the switch-cost reducing effect of the pre-cue that reduces the number of candidate tasks from four to two.

## Materials and Methods

### Participants

Twenty-one women and 5 men with a mean age of 23.3 years (range: 19–29) participated. All had normal or corrected-to-normal vision (contact lenses were not allowed). The study was approved by the local ethics committee of the Leibniz Research Centre for Working Environment and Human Factors. All participants gave their written informed consent for study participation.

### EBR Measurement

For recoding eye movements, a BrainVision QuickAmp (Brain Products^TM^ GmbH, Germany) system with two vertical (one upper, one lower) Ag-AgCl electrodes was used. Participants were comfortably seated in front of a blank poster with a fixation cross at eye level with a distance of about 1 m. They were instructed to look at the cross in a relaxed state without moving their head or activating facial muscles to avoid EOG artifacts. During measurement, the experimenter left the room. As EBR is supposed to be stable during the day but to increase in the evening (08:30 p.m., Barbato et al., 2000), data were not collected after 5 p.m. EBR was measured three times for 6 min each, before the beginning of the task switching experiment (t1), after seven experimental blocks (t2) and at the end of the session (t3). The first measurement was meant to obtain a measure of interindividual EBR differences unaffected by the upcoming task and to provide a baseline for the following measures. Raw measurements were converted to standardized EBRs (blinks/min).

The whole experimental session took place in a windowless room with constant lightning conditions, avoiding dazzling during EBR measurement as well as screen reflections during the task switching procedure.

### Stimuli, Tasks, and Apparatus

Imperative stimuli consisted of combinations of one digit from range 1–9 (excluding 5) and one of the letters A, B, E, G, N, O, S, and U. Each stimulus was about 7 mm in height and 4 mm in width. Letters and digits were presented side by side, their position chosen randomly in every trial. Task-relevant stimuli were equally distributed across the tasks, the other (to be ignored) stimulus was chosen at random in every trial. Task cues consisted of a dark blue square, diamond, circle, or triangle surrounding the position of the imperative stimulus with a size of about 70 mm × 70 mm. Participants switched among four tasks. Two of them were numerical judgment tasks, one regarding the magnitude (smaller vs. larger than five) and one regarding the parity of the digits. The magnitude task was indicated by the diamond, the parity task by the circle. In the two letter tasks, letters had to be judged regarding their position in the alphabet (first or second half), indicated by the triangle, or whether it was a vowel or a consonant, indicated by the square. To reduce the set of candidate tasks from four to two, small pre-cues were presented in a row above (square and triangle) and below (circle and diamond) the position of the imperative stimulus with a size of about 15 mm × 15 mm each. Initially, all four pre-cues were colored gray (no reduction of the set of candidate tasks), with two of them turning dark blue in half of the trials (reduction condition).

Stimuli were presented centrally on a 17″ monitor on light-gray background. Viewing distance was not restricted but amounted to approximately 60 cm. Responses were made by pressing the ‘y’-key of a German QWERTZ-keyboard for small and even digits as well as for vowels and letters from the first half of the alphabet and the ‘-‘-key for large and odd digits, for letters from the second half and for consonants.

### Task Switching Procedure

At the beginning of the experiment, participants were provided with on-screen instructions in which the tasks and the meaning of the cues were explained. Instructions emphasized speed as well as accuracy. Participants were informed that at the beginning of each trial, the four pre-cues would be visible in gray color above and below the position of the imperative stimulus and that in some trials, two of the pre-cues would turn blue, indicating that one of the two tasks whose pre-cues changed color would be the relevant one in the next trial. Participants were advised to use this information to prepare especially for the two remaining candidate tasks.

The probability of each task to be the relevant one in the next trial was 0.25, which corresponds to an overall repetition proportion of 0.25 (cf. Kleinsorge and Scheil, 2015, Exp. 2). In half of the trials, no pre-cue was presented, meaning that no tasks could be excluded because none of the cues symbolizing each of the four tasks changed color. For the other trials, two of the cues turned blue and remained so for 1,500 ms. This change of color provided the pre-cue. Pre-cues indicated each combination of two candidate tasks with equal probability. Thus, the pre-cue increased the probability of two of the tasks to 0.50. Pre-cues were shown until the presentation of the task cue. For the task cue, two CTIs (cue-target intervals) of 0 and 800 ms were employed. That is, the task cue could either be presented in advance or concurrently with the imperative stimulus. The duration of the CTI was evenly and pseudo-randomly distributed across the tasks and across the two levels of pre-cue presentation. The response-stimulus interval (RSI) was set to 2,500 ms. In case of an error, error feedback was presented for additional 1,000 ms; in case of reaction times (RTs) slower than the RT deadline of 2,500 ms, RT feedback was presented for additional 1,000 ms. Stimuli and task cue remained visible until the participant’s reaction or until RT deadline was reached. The experiment consisted of 14 blocks of 96 trials each. [A more detailed description can be found in Kleinsorge and Scheil (2015)]. Between the blocks, participants were allowed to rest and to continue the experiment in a self-paced manner in order to minimize fatigue effects. The whole session lasted for about 2 h.

## Results

The analysis of the data proceeded in several steps. In a first step, mean individual RTs and error rates (ERs) were analyzed as a function of Pre-Cue (no pre-cue vs. pre-cue), CTI (0 vs. 800 ms), and Task Transition (repetition vs. switch). Then, we analyzed mean individual EBRs during the course of the experiment. Subsequently, we augmented the preceding analyses by including additional between-participants factors representing interindividual differences in EBRs. Specifically, we subdivided our sample of participants by median splits computed on the basis of (a) initial EBRs measured at the beginning of the experiment (EBR_t1_), (b) changes of EBRs during the first half of the experiment (EBR_t1_ – EBR_t2_), and (c) changes of EBRs during the second half of the experiment (EBR_t2_ – EBR_t3_). Whereas initial EBRs were taken as a measure of overall interindividual differences in dopamine level, changes of EBRs across phases of the experiment were taken as measures of interindividual differences in adapting to the task in terms of dopamine responses. Changes between Phases 1 and 2 should reflect mainly functional adaptations in terms of dealing with (certain aspects of) the task, whereas changes between Phases 2 and 3 probably also reflect processes of saturation and fatigue.

### Overall Analyses of Task Performance

The ANOVA of mean individual RTs as a function of Pre-Cue (no pre-cue vs. pre-cue), CTI (0 vs. 800 ms), and Task Transition (repetition vs. switch) yielded significant main effects of all factors (cf. **Table 1**). The presentation of a pre-cue that reduced the number of candidate tasks from four to two decreased RT from 1,041 ms to 982 ms, *F*(1,25) = 86.66, MSe = 2,119, $\eta_{p}^{2}$ = 0.78. A CTI of 800 ms decreased RT to 802 ms, as compared to a CTI of 0 ms (1,222 ms), *F*(1,25) = 661.71, MSe = 13,852, $\eta_{p}^{2}$ = 0.96. Task switches went along with a mean RT of 1,072 ms, as compared to 951 ms with task repetitions, *F*(1,25) = 86.50, MSe = 8,859, $\eta_{p}^{2}$ = 0.78. All main effects were significant at *p* < 0.001.

**Table 1:** Mean reaction times (RT) (ms) and error rate (ER) (%) as a function of pre-cue (no presentation vs. presentation of a pre-cue), CTI (0 ms vs. 800 ms), and task transition (Repetition vs. Switch).

	No pre-cue		With pre-cue		
	CTI 0 ms	CTI 800 ms	CTI 0 ms	CTI 800 ms	*M*
**RT**					
Task repetition	1184 (26)	753 (29)	1151 (28)	716 (25)	951 (25)
Task switch	1329 (28)	899 (30)	1223 (29)	839 (30)	1072 (27)
*M*	1257 (26)	826 (28)	1187 (28)	777 (27)	
**ER**					
Task repetition	7.5 (1.0)	5.0 (0.7)	7.3 (1.0)	4.2 (0.7)	6.0 (0.8)
Task switch	9.8 (1.2)	7.6 (1.0)	8.0 (0.9)	6.7 (0.9)	8.0 (1.0)
*M*	8.7 (1.0)	6.3 (0.7)	7.6 (0.9)	5.5 (0.7)	

*SEM are given in parentheses.*

Decreasing the number of candidate tasks from four to two decreased switch costs from 145 to 98 ms, *F*(1,25) = 22.80, MSe = 1,275, *p* < 0.001, $\eta_{p}^{2}$ = 0.48. Despite the tremendous benefit that the presentation of a task cue provided with respect to mean RT, mean switch costs were lower with a CTI of 0 ms (108 ms) as compared to a CTI of 800 ms (134 ms), *F*(1,25) = 5.71, MSe = 1,605, *p* < 0.05, $\eta_{p}^{2}$ = 0.19. As revealed by a significant interaction of Pre-Cue, CTI, and Task Transition, *F*(1,25) = 7.06, MSe = 1,110, *p* < 0.05, $\eta_{p}^{2}$ = 0.22, this increase of switch costs by an increase of the CTI was confined to conditions with only two candidate tasks. When there was no pre-cue that restricted the number of candidate tasks, switch costs were nearly the same for conditions with a CTI of 0 ms (145 ms) and 800 ms (146 ms). However, when a pre-cue reduced the number of candidate tasks from four to two, a CTI of 0 ms was associated with a switch cost of 72 ms, which increased to 123 ms with a CTI of 800 ms.

The corresponding ANOVA of ERs only yielded significant main effects of all three factors. A reduction of the number of candidate tasks from four to two decreased ER from 7.5 to 6.6%, *F*(1,25) = 13.06, MSe = 0.00035, *p* < 0.01, $\eta_{p}^{2}$ = .34. A CTI of 0 ms was associated with a mean ER of 8.2%, as compared to a mean ER of 5.9% with a CTI of 800 ms, *F*(1,25) = 52.55, MSe = 0.00052, *p* < 0.01, $\eta_{p}^{2}$ = 0.68. Task switches increased ER to 8.0%, as compared to an ER of 6.0% with task repetitions, *F*(1,25) = 10.12, MSe = 0.0022, *p* < 0.01, $\eta_{p}^{2}$ = 0.29.

As can be seen from **Table 1**, there was no hint that the performance data were compromised by speed-accuracy trade-offs.

### Analysis of EBRs

Mean EBRs (blinks per minute) amounted to 19.87 (SD: 13.13) at t1, to 21.72 (SD: 14.14) at t2, and to 24.17 (SD: 13.89) at t3. The increase of EBRs during the course of the experiment was significant, *F*(2,50) = 5.05, MSe = 24.01, *p* < 0.05, $\eta_{p}^{2}$ = 0.17. Newman–Keuls *post hoc* tests revealed that EBRs at t1 differed significantly from EBRs at t3 (*p* < 0.01), whereas the difference between t1 and t2 was not significant (*p* > 0.15). The difference between t2 and t3 was marginally significant (*p* < 0.08). Individual EBRs were highly intercorrelated, with *r*’s ranging between 0.82 (t1, t3) and 0.92 (t2, t3).

### Analyses of Task Performance Including Individual Differences in EBRs

#### EBRs at _t1_

Subdividing our sample of participants according to their EBRs at t1 by a median split (median: 18.08) and entering this between-participants factor Initial EBR into the analyses of RTs and ERs as a function of Pre-Cue (no pre-cue vs. pre-cue), CTI (0 vs. 800 ms), and Task Transition (repetition vs. switch) yielded no significant interactions including the factor Initial EBR in the analysis of RTs (all *p*’s > 0.25).^1^

**FIGURE 1:**
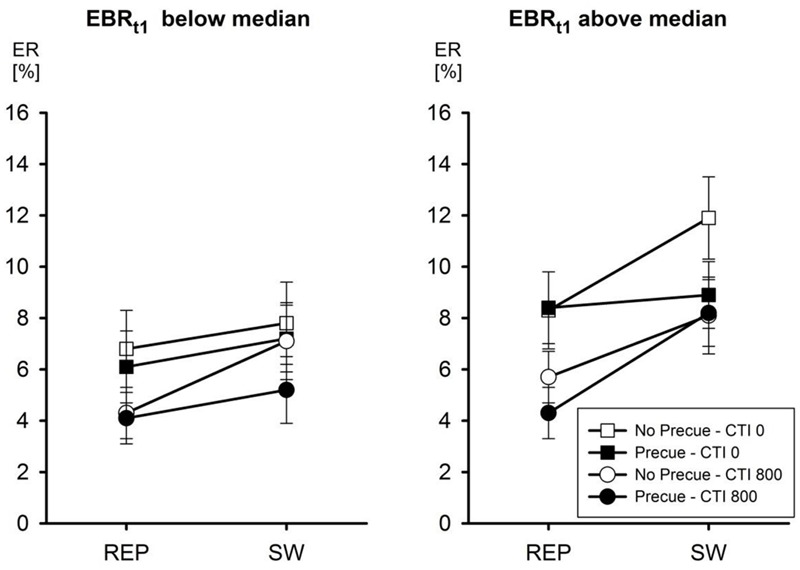
**Mean error rate (ER) as a function of eye blink rate (EBR) at _t1_, Pre-Cue, CTI, and task transition.** Error bars represent SEM.

In the analysis of ERs, however, Initial EBR entered into a significant third-order interaction of all four factors, *F*(1,24) = 8.73, MSe = 0.0004, *p* < 0.01, $\eta_{p}^{2}$ = 0.27. This interaction is depicted in **Figure 1**. In line with the main focus of the present study, we interpret this interaction regarding the effect of decreasing the number of candidate tasks from four to two, that is, the effect of Pre-Cue. For participants with an Initial EBR below the median, the presentation of a pre-cue had only a negligible effect on ERs. Only with a CTI of 800 ms there was a tendency that the reduction of the number of tasks decreased switch costs (from 2.7 to 1.1%), but this was statistically not significant (all p’s > 0.15 according to Newman–Keuls *post hoc* tests). In contrast, for participants with an Initial EBR above the median the presentation of a pre-cue significantly reduced the ER associated with a task switch from 12.0 to 8.9 with a CTI of 0 ms, *p* < 0.001, but not with a CTI of 800 ms (*p* > 0.8). Pre-Cue did not affect ERs with task repetitions at any level of CTI (*p*’s > 0.25).

#### EBR_t1_ – EBR_t2_

In a next step, we replaced the between-participants factor Initial EBR by a factor based on the individual differences in EBRs between t1 and t2. Specifically, we subtracted for each participant the EBR measured at t2 from the EBR measured at t1, and subsequently subdivided our sample by a median split according to this difference (Median: -1.29). Thus, there was a median increase of mean EBR from t1 to t2 of 1.29 blinks per minute. Note that the split of our sample along the median is almost identical to a split in terms of an absolute increase vs. decrease of EBRs across the two times of measurement. In fact, the latter way of splitting participants into subgroups would have resulted in only one participant being assigned to another group, with this difference having no substantial effect on our main results. The differences EBR_t1_ – EBR_t2_ correlated only weakly with the EBRs measured at t1 (*r* = 0.21, n.s.).

**FIGURE 2:**
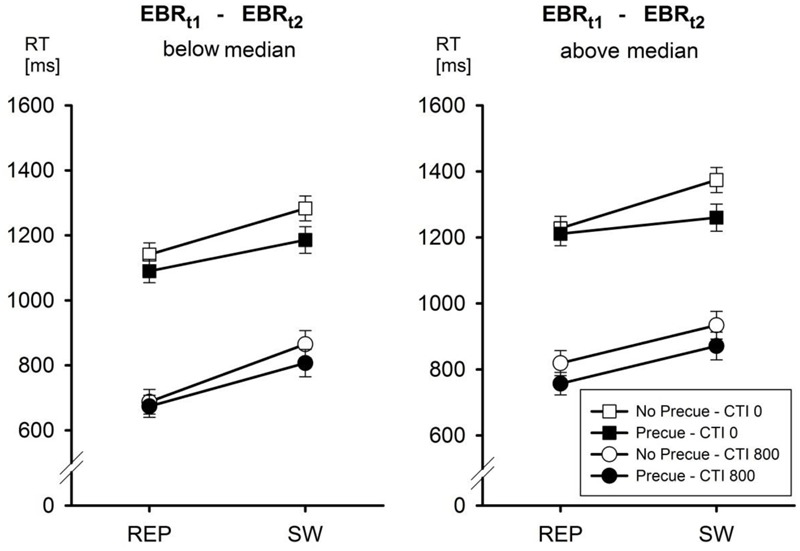
**Mean reaction time (RT) as a function of EBR _t1-t2_, Pre-Cue, CTI, and task transition.** Error bars represent SEM.

Entering the between-participants factor EBR_t1_ – EBR_t2_ into the analyses of RTs and ERs as a function of Pre-Cue (no pre-cue vs. pre-cue), CTI (0 vs. 800 ms), and Task Transition (repetition vs. switch) yielded the following picture (cf. **Table 2**). In the analysis of RTs, the only significant interaction involving EBR_t1_ – EBR_t2_ was the third-order interaction of all four factors, *F*(1,24) = 8.2, MSe = 862, *p* < 0.01, $\eta_{p}^{2}$ = 0.25. This interaction is depicted in **Figure 2**. This interaction is based on the observation that the second-order interaction Pre-Cue × CTI × Task Transition was significant [*F*(1,12) = 17.85, MSe = 834, *p* < 0.01, $\eta_{p}^{2}$ = 0.60] only in the group of participants with a EBR_t1_ – EBR_t2_ difference above the median, that is, for participants tending to decrease their EBR in the first half of the experiment. In contrast, in the group of participants with an EBR_t1_ – EBR_t2_ difference below the median this interaction was far from significant, *F* < 1. Newman–Keuls *post hoc* tests indicated that this pattern was due to the fact that in the group of participants with a EBR_t1_ – EBR_t2_ difference above the median, reducing the number of candidate tasks from four to two reduced switch costs only with a CTI of 0 ms (from 146 to 49 ms, *p* < 0.001), but not with a CTI of 800 ms (switch costs 115 vs. 114 ms with no pre-cue vs. pre-cue). In contrast, in the group of participants with a EBR_t1_ – EBR_t2_ difference below the median, switch costs were reduced by the Pre-Cue both with a CTI of 0 ms (142 vs. 95 ms, *p* < 0.05) and with a CTI of 800 ms (177 vs. 133 ms, *p* < 0.05).

**Table 2:** Mean RT (ms) and ER (%) as a function of EBR_T1-T2_ (below vs. above median), pre-cue (no presentation vs. presentation of a pre-cue), CTI (0 ms vs. 800 ms), and task transition (Repetition vs. Switch).

		EBR_T1-T2_ below median				EBR_T1-T2_ above median			
		No pre-cue		With pre-cue		No pre-cue		With pre-cue	
		CTI 0 ms	CTI 800 ms	CTI 0 ms	CTI 800 ms	CTI 0 ms	CTI 800 ms	CTI 0 ms	CTI 800 ms
RT	Task repetition	1141 (36)	688 (38)	1090 (36)	674 (34)	1228 (36)	819 (38)	1211 (36)	757 (34)
	Task switch	1283 (38)	865 (42)	1186 (41)	807 (42)	1374 (38)	934 (42)	1260 (41)	871 (42)
	*M*	1212 (35)	776 (38)	1138 (37)	741 (37)	1301 (35)	876 (38)	1236 (37)	814 (37)
ER	Task repetition	8.8 (1.4)	4.4 (1.0)	7.3 (1.4)	4.7 (1.0)	6.2 (1.4)	5.6 (1.0)	7.2 (1.4)	3.7 (1.0)
	Task switch	10.1 (1.7)	8.0 (1.5)	8.0 (1.4)	7.4 (1.3)	9.5 (1.7)	7.2 (1.5)	8.0 (1.4)	6.0 (1.3)
	*M*	9.5 (1.4)	6.2 (1.0)	7.7 (1.3)	6.1 (1.0)	7.9 (1.4)	6.4 (1.1)	7.6 (1.3)	4.9 (1.0)

*SEM are given in parentheses.*

In the corresponding analysis of ERs, the only interaction involving the between-participants factor EBR_t1_ – EBR_t2_ was the second-order interaction EBR_t1_ – EBR_t2_ × Pre-Cue × CTI, *F*(1,24) = 8.37, MSe = 0.00035, *p* < 0.01, $\eta_{p}^{2}$ = 0.26. This interaction was due to the fact that in the group of participants with a EBR_t1_ – EBR_t2_ difference below the median, reducing the number of candidate tasks reduced ER more with a CTI of 0 ms (from 9.5 to 7.7%) than with a CTI of 800 ms (from 6.2 to 6.1%). In the group of participants with an EBR_t1_ – EBR_t2_ difference above the median, this pattern was reversed (7.9 vs. 7.6% with CTI 0, 6.4 vs. 4.9% with CTI 800).

#### EBR_t2_ – EBR_t3_

In a final step, we replaced the between-participants factor based on the individual differences in EBRs between t1 and t2 by a factor based on a median split of the individual differences in EBRs between t2 and t3 (Median: -0.96). As with the median split regarding the differences in EBRs between t1 and t2, this median is close to zero. Splitting participants into subgroups in terms of an absolute increase vs. decrease of EBRs across the two times of measurement would have resulted in two participants being assigned to another group, with this difference having no substantial effect on our main results The differences EBR_t2_ – EBR_t3_ correlated only weakly with the differences EBR_t1_ – EBR_t2_ (*r* = -0.17, n.s.).

Entering the new between-participants factor EBR_t2_ – EBR_t3_ into the analysis of RTs yielded only one significant interaction involving EBR_t2_ – EBR_t3_. This was the interaction EBR_t2_ – EBR_t3_ × CTI, *F*(1,24) = 4.95, MSe = 11,960, *p* < 0.05, $\eta_{p}^{2}$ = 0.17. This was based on the observation that the reduction of RTs induced by a CTI of 800 ms was more pronounced in the group with a EBR_t2_ – EBR_t3_ difference below the median (1,191 vs. 737 ms), as compared to the group with a EBR_t2_ – EBR_t3_ difference above the median (1,253 vs. 867 ms). The corresponding analysis of ERs yielded no significant interaction involving EBR_t2_ – EBR_t3_, all *p*’s > 0.12.

## Discussion

The results of the present study can be summarized as follows. First, apart from replicating basic task switching effects (switch costs, effect of CTI), we replicated the main finding of Kleinsorge and Scheil (2015). We again observed that the presentation of a pre-cue that reduced the number of candidate tasks from four to two mainly affected task switches and therefore reduced switch costs substantially. One deviation from the original findings of Kleinsorge and Scheil (2015) consists of the observation of a significant second-order interaction of Pre-Cue, CTI, and Task Transition in the present study. However, as will be discussed below, the observation of this interaction was restricted to a subgroup of participants of the present study and not observed in another subgroup.

Coming to the effects of interindividual variations of EBRs on task switching performance with the current double-cue paradigm, overall differences in EBRs as measured at the beginning of the experiment had only a minor effect that was restricted to accuracy. Specifically, our observations suggest that participants with an initial EBR above the median were better able to use the pre-cue to increase accuracy, with this effect being restricted to task switches with CTI of 0 ms. This finding is in line with the assumption that higher baseline levels of dopamine facilitate task switching (cf. Jongkees and Colzato, 2016). Furthermore, it seems that one specific process being facilitated by relatively high levels of dopamine is the restriction of the repertoire of candidate actions in line with dynamically changing situational demands, perhaps by adjusting the relative amount of lateral inhibition among action alternatives.

Whereas the effect of overall differences in EBRs on task performance was rather restricted under the current conditions, changes of EBR during the first part of the experiment had a more tremendous impact. In particular, participants who tended to increase their EBR when dealing with the task made use of the pre-cue irrespective of the level of CTI, whereas participants who tended to decrease their EBR seemed to follow a more disjunctive strategy in that the effects of the pre-cue were different when a task cue was available than when it was not^2^. With no task cue (CTI = 0), the presentation of a pre-cue affected task switches much more than task repetitions, whereas with a task cue task repetitions and switches were affected by the pre-cue to the same degree. This observation suggests that for this group of participants, the strategy of using the pre-cue was influenced by the presentation of a task cue, which happened after encoding of the precue should have taken place. A possible explanation for this somewhat counterintuitive assumption relates to the temporal features of the different trial types. Specifically, trials in which both types of cue information were presented in advance are characterized by an onset of the pre-cue only 200 ms after the beginning of the trial. That is, if the initial display changes immediately after trial onset, participants can infer that in this trial, not only a pre-cue but also a task cue will be presented. This could have led participants to use the information of the pre-cue only superficially and to rely to a larger degree on the information given by the task cue. In contrast, if nothing happens immediately after trial onset, participants are not able to distinguish between the other three conditions in advance. This seems to be a methodological shortcoming, however, the alternative solution would have been a longer presentation of the pre-cue in trials with a CTI of 0 ms, which would have resulted in a confound of both intervals or, alternatively, the use of different intertrial intervals which also would have conveyed predictive information about the upcoming cuing procedure. In any case, it seems that differences in the change of EBRs while adapting to the task were associated with different strategies of cue use, with participants tending to decrease their EBR being more focused on the task cue that unambiguously specified the upcoming task.

Kleinsorge and Scheil (2015) interpreted the switch-cost reducing effect of the pre-cue in terms of a change in the way a task is selected. Specifically, we proposed that a selection among only two candidate tasks is facilitated by an establishment of antagonistic constraints among the two tasks that enables task selection based on any perceptually available feature that discriminates between the two tasks. This is possible because any evidence favoring one of the tasks is at the same time evidence against the other task. In contrast, when selecting one of four tasks, evidence against one of the tasks does not directly translate into evidence in favor of one of the remaining three candidate tasks. This line of reasoning converges upon the assumption that the effect of the pre-cue is brought about by enhancing inhibition among competing tasks, a process that is probably implemented by the striatal D2 system (cf. Keeler et al., 2014). Based on the assumption that an increase of EBRs reflects increased reliance on D2 mediated processing (cf. Jongkees and Colzato, 2016), observing a more consistent effect of the pre-cue across levels of CTI in the subgroup of participants with a EBR_t1_ – EBR_t2_ difference below the median (indicating an increased EBR) therefore makes sense. In particular, it seems that these participants consistently exploited the pre-cue to adjust the level of inter-task competition in a way that facilitated task switching induced by the task cue.

In contrast, participants who tended to decrease their EBR during the first part of the experiment (EBR_t1_ – EBR_t2_ difference above the median) exhibited a pre-cue induced reduction of switch costs only when no task cue was presented. This suggests that when a task cue was (expected to be) available, these participants selected the relevant task in a more ‘direct’ manner (possibly more reliant on D1 mediated processing) that was less reliant on inhibitory connections among competing tasks. In this case, the task cue may directly trigger the retrieval of the task with the strongest reward association, which is likely to be the currently relevant one.

Of course, at present the foregoing considerations are in large part speculative. However, we find it remarkable that the EBR-based effects we observed concern mainly the effects of pre-cue, that is, the experimental variation that we supposed to be susceptible to D2 mediated interindividual variation on *a priori* grounds, as outlined in the introduction. What is somewhat surprising is the observation of EBR-related effects mainly in terms of changes of EBRs rather than their overall level. This suggests that changes in EBRs may constitute an as long neglected marker of interindividual differences in adapting to tasks demands that place a burden on processes of task (or action) selection. At present, changes in EBRs are mainly considered as markers of fatigue (EBR increase, cf. Barbato et al., 2007; McIntire et al., 2014). When measured on-task, EBR decreases and blink suppression are positively correlated with task difficulty (cf. Oh et al., 2012; Wascher et al., 2015). However, our measure of EBR changes as a function of adaptation to task demands lies somewhere between the more global measures of state-dependent EBR changes as indictors of fatigue and the temporarily more fine-grained measures of blink suppression during more demanding phases of task performance. To the best of our knowledge, our study is the first to provide evidence that EBR changes in a time range of about 1 h are predictive of a very specific aspect of processing in a task switching context, namely the use of foreknowledge that allows for a proactive restriction of the number of alternative task options. Of course, this novelty of our results implies also a need for replication of this kind of relationship.

Overall, our findings support the assumption of an intimate link between dopaminergically modulated processes and cognitive flexibility. Although the intricacies of this link are only beginning to be understood, the double-cue procedure employed in the present experiment promises to serve as a tool to distinguish between control processes related to task switching that are differentially affected by different (possibly D1 vs. D2 mediated) dopaminergic projections. On a functional level, these processes may differ with respect to the degree by which they rely on inhibition among competing tasks (like an implementation of antagonistic constraints) vs. direct activation of a particular task based on the availability of an unambiguous task cue.

## Author Contributions

TK and JS designed the experiment, analyzed the data, and wrote the article.

## Conflict of Interest Statement

The authors declare that the research was conducted in the absence of any commercial or financial relationships that could be construed as a potential conflict of interest.

## Footnotes

1 The use of median splits has sometimes been criticized for a loss of information and/or a higher risk of statistical errors. However, as shown by Iacobucci et al. (2015a,b), these concerns are most often unwarranted when a single variable based on a median split is combined with orthogonal experimental variations that are uncorrelated with the median-split based factor. Furthermore, our study is based on the replication of a complex factorial design that lends itself to an analysis based on ANOVA in a straightforward manner, which in this case also facilitates the communication of the results as compared to regression-based approaches.

2 When analyzing this pattern in terms of correlations, we observed a positive correlation of *r* = 0.36 (*p* < 0.08) between the individual differences in EBR changes between t1 and t2 and the reduction of switch costs as a function of the pre-cue with a CTI of 0 ms, but a negative correlation of *r* = -0.37 (*p* < 0.07) between these measures with a CTI of 800 ms. While these observations corroborate our conclusion that the interaction of the effects of the two types of cues was modulated by changes in EBRs, we think that a selective comparison of single correlations across particular factorial combinations within our rather complex experimental design provides no comprehensive analytical strategy to capture our main findings.
